# Survey to Determine Perceptions and Practices in Contact Lens Use and Identify Key Features of Safe Use Education

**DOI:** 10.4274/tjo.60465

**Published:** 2018-12-27

**Authors:** Tomris Şengör, Sanem Alkibay, Ayşegül Ermeç Sertoğlu, Sevda Aydın Kurna

**Affiliations:** 1 Independent Practitioner, İstanbul, Turkey; 2 Gazi Üniversity Faculty of Economics and Administrative Sciences, Department of International Trade, Ankara, Turkey; 3 Fatih Sultan Mehmet Training and Research Hospital, Ophthalmology Clinic, İstanbul, Turkey

**Keywords:** Contact lens consumer trends, education, message contents, public health

## Abstract

**Objectives:**

To identify consumers’ tendencies regarding contact lens (CL) use in order to develop recommendations for messages to include in education for safe CL use and in public awareness campaigns.

**Materials and Methods:**

Subjects living in Ankara, Turkey who used eyeglasses and/or contact lenses due to refractive error were included in the study. CL users’ reasons for choosing CLs for vision correction, CL-related problems they encountered, and their perceptions regarding safe CL use education and regular ophthalmologic follow-up visits were evaluated using a survey completed by 917 participants.

**Results:**

In total, 836 survey forms were included in the analysis. Most of the participants were female (59.6%), university students (91.4%), and 18-30 years old (68.9%). According to the survey results, 64.6% of eyeglass users stated that they had never tried CLs, while 17.7% reported using CLs regularly. Most of the participants (61.7%) said they visit an ophthalmologist only when they needed, while 33.1% claimed to attend regular follow-up. When all participants were considered, the level of satisfaction with glasses was 3.11 out of 5, while CL users reported satisfaction of 4.15 out of 5. Most (78.6%) of the CL users said they started using CL by their own initiative, most commonly due to a dislike of eyeglasses. The most frequent complaint from CL users was dry eye and discomfort in the evening. The most common source of CL use education was ophthalmologists (55.5% of the participants), followed by opticians (28.2%).

**Conclusion:**

Incorrect and inappropriate information on CL usage may lead to problems that can threaten eye health. The results of our study suggest that providing accurate information through concise messages in physician-supervised education and raising awareness through the media may be beneficial to public health. Therefore, we identified messages about CL usage and quality of life, safety, and the rules for proper use.

## Introduction

Contact lenses (CLs) are temporary prostheses placed on the eye for optical, aesthetic, or therapeutic purposes. Glasses have been the most widely used tool for correcting refractive errors throughout human history. In terms of optical properties, because the lens sits 10-12 mm in front of the eye, glasses have a limiting effect on field of vision and may cause alterations in image size and deviations that reduce the quality of vision, particularly at high diopters. Unlike glasses, CLs correct refractive errors at the ocular surface, which helps widen field of vision, reduce spheric and chromatic aberrations and distortions compared to glasses, improve image quality, and eliminate the esthetic issues associated with glasses. CLs are especially preferred by younger populations because of this optical and esthetic superiority.^1,2,3^

Numerous studies have shown that CLs can lead to serious problems that threaten ocular health if certain basic guidelines of use are not observed, particularly those concerned with cleaning.^4,5,6,7,8^ It is crucial to raise social awareness regarding the health problems that may be caused by CLs that are purchased without a prescription and used without the proper examination, practical training, and trial provided by an ophthalmologist.^9,10^ In the present study, quantitative and qualitative research methods were used in the target population to conduct a detailed analysis of this public health issue, and the results were used to create key messages that can be used in physician-delivered user education and to raise social awareness through mass media.

## Materials and Methods

Ethics committee approval was obtained and the study was conducted in compliance with the ethical rules for human research set forth in the Declaration of Helsinki. All participants included in the survey provided written informed consent.

This study was exploratory research employing both quantitative and qualitative research methods to identify user tendencies related to CL use.^9,10^

In the first phase of the study, survey preparation, 35 randomly selected CL users were interviewed in 6 focus groups. For each focus group, the interview was planned to last at least 1 hour. The survey questions were prepared based on the information obtained from these interviews.

For the survey, a sample group representative of the general population was selected from among the residents of the district of Çankaya in Ankara, Turkey who used glasses and/or CLs for refractive correction. A total of 917 people participated in the survey. Participants who had undergone ocular surgery in the previous 3 months or were found to have any active eye disease were excluded from the study. As a result, 836 survey forms were included in the analysis.

### Statistical Analysis

Descriptive statistics (percentage, mean, standard deviation) were used to evaluate the data collected. Based on the calculated mean values, we evaluated concepts such as level of satisfaction or concern about the use of CLs and glasses and the CL-related problems encountered by CL users. Weighted total values were calculated and interpreted using rank data in order to determine the relative significance of the reasons for CL avoidance and non-CL users’ concerns regarding CL use.

## Results

The study findings are presented below under two headings.

### 1. Focus Group Interviews

A focus group interview is a carefully planned form of discussion/interview performed with a small group led by a moderator in order to obtain detailed information and elicit opinions about a topic defined by the researcher.^11^

A questioning route was used, with all questions prepared in full prior to the interviews. After conducting 6 focus group interviews with a total of 35 glasses/CL users, the frequency and repetition rates of the participants’ answers were used to restructure the response options for the survey questions.

### 2. Survey Results

The respondents’ demographic features, duration and status of glasses and CL use, and frequency of ophthalmologist visits were evaluated (Table 1). Of the survey respondents, 59.6% were women and 40.4% were men, and most were high school graduates and university students (91.4%). The majority (68.9%) of participants were 18-30 years old; most had a middle to high income level.

Duration of glasses use was >1 year for the majority of respondents and >10 years in 26.6% of the respondents. Nearly two-thirds (64.6%) of the respondents said they had never used CLs, while 17.7% used CLs continuously, 10.8% used CLs intermittently, and 6.9% had used CLs previously but since stopped. Many (61.7%) of the respondents reported visiting an ophthalmologist when necessary, whereas 33.1% stated that they attended regular follow-up.

When respondents were asked to rate their satisfaction with the use of glasses and CLs (on a scale of 1 to 5), mean satisfaction among the entire group was slightly above neutral for glasses (3.11±1.18), while CL users reported a much higher mean satisfaction level for CLs (4.15±0.73) (Table 2).

When evaluated separately based on CL use, the group with no CL experience was more satisfied with glasses. In contrast, the respondents who reported continuous CL use were least satisfied with glasses, as expected. Degree of satisfaction with CL use also differed between continuous and intermittent users, with continuous users reporting greater satisfaction (Table 3).

The survey included a question for non-CL users (both those who used them previously and those who never used them) regarding their reasons for avoiding CL use. Table 4 shows the importance levels of the possible causes determined according to the focus group interviews. The most important reason for avoiding CL use was the belief that CL use is difficult. The second and third reasons were the convenience of wearing glasses, and the opinion that CLs harm the eyes.

All of the non-CL users were also asked to indicate their level of concern about the difficulties that can be experienced while using CLs. The potential difficulties that have been or may be experienced while using CLs were identified and listed after the focus group interviews (Table 5). Mean values indicate that the biggest concern is the possibility of eye infection due to CL use. Other major problems included fear of the lens sticking to the eye or experiencing a stinging/foreign body sensation. Another potential problem mentioned during the focus group interviews was difficulty with near vision while wearing CLs, but this was not a concern for the non-CL users. Similarly, the beliefs that CLs may lead to refractive error progression, cause cataracts, and prevent laser eye surgery in the future caused less concern than the mean value of 3.284.

Finally, non-CL users were asked to state the source of their concerns about CL use and rank the information sources identified in the focus group interviews based on their importance. The most important source of concerns related to CL use was personal observations, followed by information obtained from immediate social circles, and news in the printed/visual media (Table 6).

The next section of the questionnaire consisted of questions for CL users. Lens users (continuous and intermittent users) were first asked about how/why they started wearing CLs. The response options for this question were based on findings from the focus group interviews. Accordingly, most of the participants (78.6%) stated that they began wearing lenses by their own initiative and 9.2% started following recommendations from others (Table 7).

The participants were asked to rank the factors that influenced their decision to wear CLs in order of importance. The most influential reason was that they disliked and were tired of glasses. Other reasons included esthetic concerns and the inconveniences of glasses (limited vision, fogging, getting wet in the rain, etc.) (Table 8).

When asked about the difficulties they experienced, CL users’ most common problem was dry eye, followed by discomfort and stinging in the eye in the evenings as a result of wearing CLs all day long. The least common problems were ocular surface scratches, problems with near vision, and blurred vision. Eye infection, which was the biggest concern of non-CL users, had never occurred in 54.6% of the CL users. Similarly, the fear that a lens may adhere to the eye was shown to be a misconception that should be dispelled, as most CL users did not experience this problem (Table 9).

When the CL users were asked where they obtained information about how to use CLs, 55.5% reported getting the information from ophthalmologists, and the second most common source was opticians (Table 10).

## Discussion

CLs are temporary prostheses placed on the eye for optical, esthetic, or therapeutic reasons and are considered optically and esthetically superior to glasses. Similarly, our results showed that among all participants, the mean level of satisfaction was 3.11/5 for users of glasses and higher in CL users, at 4.15/5.

In spite of their advantages, preference for CLs may not be as high as expected in Turkey. In fact, 64.6% of the participants in our study said they had never tried CLs, while only 17.7% reported using CLs regularly. Based on the inclinations of the group that did not prefer CLs, their main reason for avoidance was the belief that CL use is difficult, and their main concern was the possibility of eye infection while using CLs. The fact that these well-educated participants’ primary source of concern about CL use was information acquired through their own observations or from their immediate social circles suggests a lack of access to sufficient and reliable information through proper and effective channels.

Among the CL users, 78.6% said that they started using CLs on their own initiative, mainly due to disliking glasses and wanting to stop wearing them. Other reasons included esthetic concerns about wearing glasses and the related discomfort (limited vision, fogging, getting wet in the rain, etc.).

On the other hand, CLs are in direct contact with corneal surface and eyelids. Each CL user differs in terms of occupation, environmental conditions, tear film properties, corneal gradient and diameter, and anatomical features such as interpalpebral distance and eyelid shape. Therefore, CLs should be prescribed by an ophthalmologist who can select suitable materials, surface and edge designs, and curvature radius based on variable environmental conditions and eye anatomy and physiology. In best practice, the ophthalmologist chooses a lens according to these individual variables and allows the patient to wear it for a time in order to evaluate compatibility with the ocular surface and eyelids and confirm the refractory power of the lens. After this trial period, the patient is provided a basic theoretical and practical training focusing heavily on cleaning, and finally the lens is prescribed. If CLs are used without the supervision and education provided by ophthalmologists, these important steps are neglected, which greatly increases the likelihood of complications that threaten ocular health, such as corneal ulcers.^4,5,6,7,8^ Thus, legislation governing the health care system (Law and Regulation number 5193) states that CL examination must only be done by ophthalmologist, and forbids opticians from selling CLs without a prescription. However, available data indicate that CLs are being sold without prescription and used inappropriately in Turkey.^9,10^

A survey of 443 university students conducted by Dinç et al.^9^ revealed that 47.3% of the participants received basic information about CLs from an ophthalmologist, while the rest learned this information from various sources. In addition, only 43.9% of the participants visited the ophthalmologist regularly while using CLs. Similarly, many of the participants in our study (61.7%) reported seeing an ophthalmologist only when needed, while 33.1% visited regularly. The remarkably low rates of regular follow-up in both studies indicate an important deficit.

Donshik et al.^12^ noted that nonadherence to guidelines for safe CL wear is still a major contributor to CL-related complications and discontinuation of CL use. They also emphasized that lack of information, bad habits, misconceptions, and the inadequacy of available information sources all play a role in this noncompliance.

Wu et al.^13^ evaluated noncompliant behaviors in 210 CL usersand identified hand hygiene, improper lens care, and inability to remember follow-up appointments as the main problems, noting that the ability to purchase CLs online results in unawareness regarding follow-up examinations.

As for education in CL use, we also found that only 56% of the participants in our study had received information about how to use CLs from their ophthalmologist, while the other half reported getting that information from people without adequate knowledge and authority. Consistent with previous reports, our study shows that CL users’ adherence to basic guidelines, such as attending regular follow-up and receiving practical education in CL use directly from an ophthalmologist, is far below necessary levels. These findings indicate that the public is not adequately informed and aware of these issues.

Ensuring appropriate CL use is a matter of protecting public health and enhancing social awareness. To ensure appropriate CL use, it is key to promote users’ awareness of important evidence-based and legally regulated issues related to the examination, procurement, and usage of CLs. To achieve the greatest benefit from this awareness raising, concise messages conveyed in physician-delivered education and via mass media have an important impact on the perceptions of CL users. 

Based on this and the findings of our study, the main elements to be emphasized in public awareness and user education are summarized as follows: CLs improve quality of life; CLs are not harmful to the eyes when guidelines for safe use are followed; cleaning and disinfecting are essential; dryness and stinging are common but easily solved problems; and CLs should be used under ophthalmologist supervision. To the same end, initiatives to raise public awareness were implemented by the Turkish Ophthalmological Association Contact Lens Unit as part of the Contact Lens Information Project. Using slogans of “Did you consult your eye doctor?” and “Do you follow the rules?”, the campaign yielded positive results, which is promising for the promotion of public awareness in Turkey.

## Conclusion

In conclusion, incorrect and inadequate information about CL use may result in problems that threaten eye health. Our findings suggest that disseminating accurate information and proper guidelines through concise messages in physician-provided education and raising awareness via mass media will help protect public health. Therefore, we identified message content about CL usage and quality of life, safety, and rules for proper use.

However, a noteworthy limitation of our study is that data were not collected regarding the participants’ refractive error, CL type, and whether they purchased CLs by prescription or online. Subsequent research among CL users that utilizes a survey including these details will further raise awareness of this issue.

## Figures and Tables

**Table 1:**
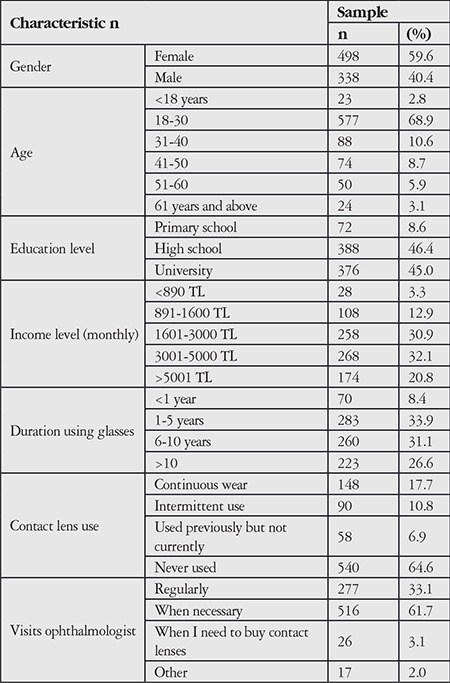
Demographic and contact lens-related data of the study participants

**Table 2:**
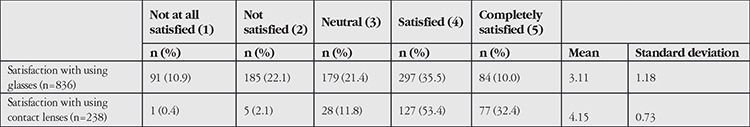
Satisfaction levels of participants using glasses and contact lenses

**Table 3:**
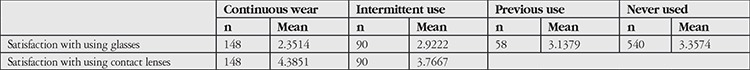
Satisfaction levels of participants using glasses and contact lenses according to their contact lens use

**Table 4:**
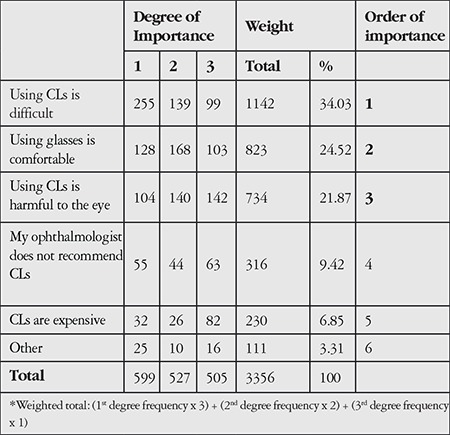
Levels of importance of participants’ reasons for contact lens avoidance

**Table 5:**
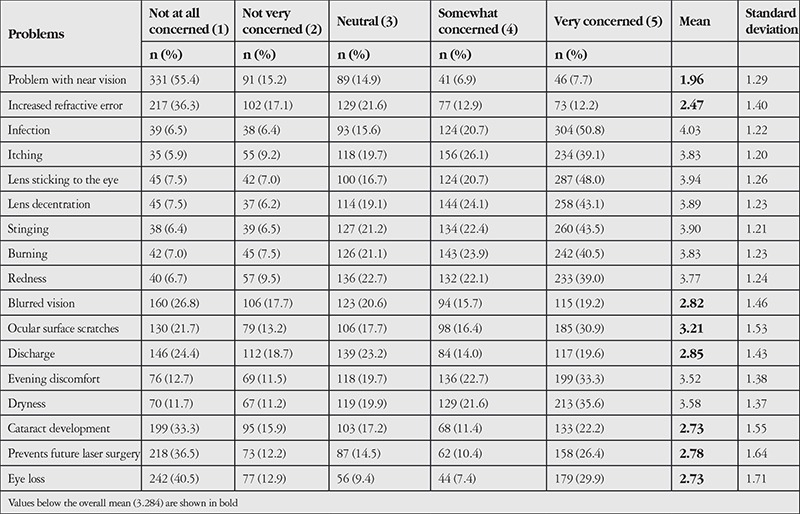
Concerns related to contact lens use among participants who did not use contact lenses

**Table 6:**
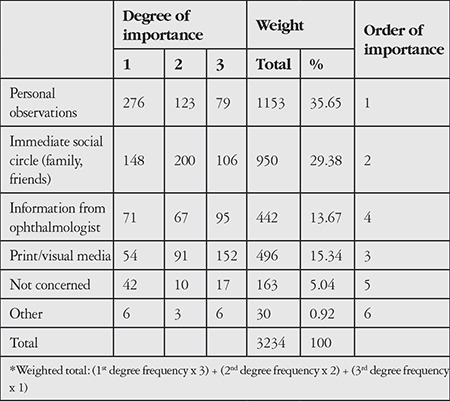
Source of concerns about contact lens use

**Table 7:**
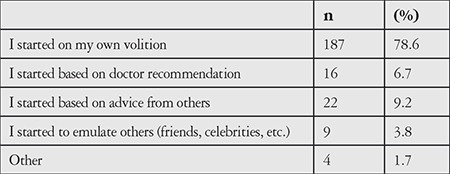
How the participants started using contact lenses

**Table 8:**
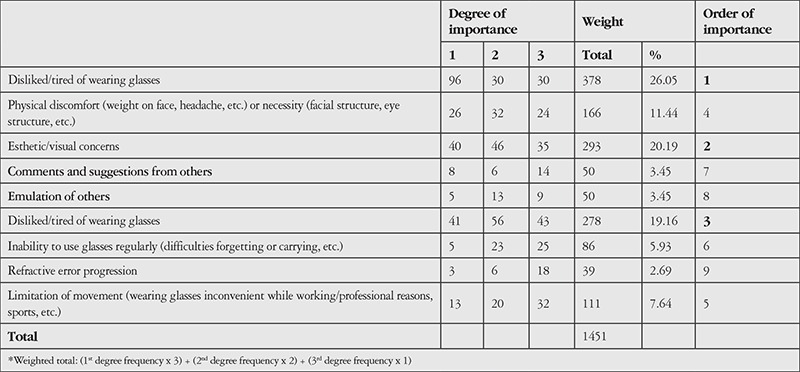
Importance of reasons influencing the decision to start using contact lenses

**Table 9:**
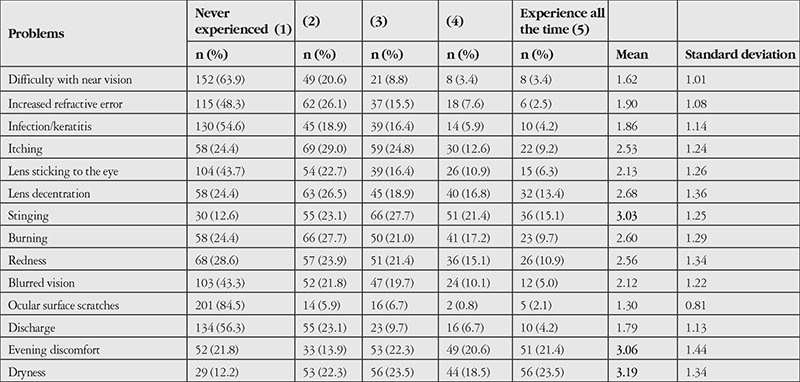
Frequency of contact lens-related problems experienced by contact lens users

**Table 10:**
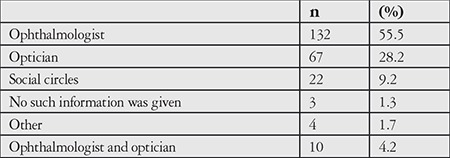
Source of information about contact lens use
